# A User-Friendly System for Mailed Dosimetric Audits of ^192^Ir or ^60^Co HDR Brachytherapy Sources

**DOI:** 10.3390/cancers15092484

**Published:** 2023-04-26

**Authors:** Laura Oliver-Cañamás, Javier Vijande, Cristian Candela-Juan, Jose Gimeno-Olmos, Mª Carmen Pujades-Claumarchirant, Juan J. Rovira-Escutia, Facundo Ballester, Jose Perez-Calatayud

**Affiliations:** 1 Servei de Radiofísica i Protecció Radiològica, Consorci Hospitalari Provincial de Castelló (CHPC), 12002 Castelló de la Plana, Spain; 2 Departamento de Física Atómica, Molecular y Nuclear, Universitat de Valencia (UV), 46100 Burjassot, Spain; 3 Unidad Mixta de Investigación en Radiofísica e Instrumentación Nuclear en Medicina (IRIMED), Instituto de Investigación Sanitaria La Fe (IIS-La Fe), Universitat de Valencia (UV), 46026 València, Spain; 4 Instituto de Física Corpuscular, Instituto de Física Corpuscular—IFIC (UV-CSIC), 46100 Burjassot, Spain; 5 Radiation Oncology Department, Hospital Clínic de Barcelona, 08036 Barcelona, Spain; 6 Unitat de Radiofísica, Servei d’Oncologia Radioteràpica, Hospital Universitari i Politècnic La Fe, 46026 València, Spain; 7 Centro Nacional de Dosimetría, 46009 València, Spain; 8 Servei de Radiofísica i Protecció Radiològica, Consorci Hospital General Universitari de València, 46014 València, Spain; 9 Hospital Clínica Benidorm, 03501 Benidorm, Spain

**Keywords:** high dose rate brachytherapy, dosimetric audit, error detection, phantom

## Abstract

**Simple Summary:**

Nowadays, the options available to perform external dosimetric audits of the high dose rate (HDR) brachytherapy treatment process are limited. In this work, we present a methodology that allows for performing dosimetric audits in this field. A phantom was designed and manufactured for this purpose. The criteria for its design, together with the in-house measurements for its characterization, are presented. The result is a user-friendly system that can be mailed to perform dosimetric audits in HDR brachytherapy on-site for systems using either Iridium-192 (^192^Ir) or Cobalt-60 (^60^Co) sources.

**Abstract:**

Objectives: The main goal of this work is to design and characterize a user-friendly methodology to perform mailed dosimetric audits in high dose rate (HDR) brachytherapy for systems using either Iridium-192 (^192^Ir) or Cobalt-60 (^60^Co) sources. Methods: A solid phantom was designed and manufactured with four catheters and a central slot to place one dosimeter. Irradiations with an Elekta MicroSelectron V2 for ^192^Ir, and with a BEBIG Multisource for ^60^Co were performed for its characterization. For the dose measurements, nanoDots, a type of optically stimulated luminescent dosimeters (OSLDs), were characterized. Monte Carlo (MC) simulations were performed to evaluate the scatter conditions of the irradiation set-up and to study differences in the photon spectra of different ^192^Ir sources (Microselectron V2, Flexisource, BEBIG Ir2.A85-2 and Varisource VS2000) reaching the dosimeter in the irradiation set-up. Results: MC simulations indicate that the surface material on which the phantom is supported during the irradiations does not affect the absorbed dose in the nanoDot. Generally, differences below 5% were found in the photon spectra reaching the detector when comparing the Microselectron V2, the Flexisource and the BEBIG models. However, differences up to 20% are observed between the V2 and the Varisource VS2000 models. The calibration coefficients and the uncertainty in the dose measurement were evaluated. Conclusions: The system described here is able to perform dosimetric audits in HDR brachytherapy for systems using either ^192^Ir or ^60^Co sources. No significant differences are observed between the photon spectra reaching the detector for the MicroSelectron V2, the Flexisource and the BEBIG ^192^Ir sources. For the Varisource VS2000, a higher uncertainty is considered in the dose measurement to allow for the nanoDot response.

## 1. Introduction

In the high dose rate (HDR) brachytherapy field, multiple sources of error may be found. Some examples are the calibration of the well-type chamber used to measure the reference air kerma rate (RAKR), the source position, the irradiation time, or the image reconstruction [1]. However, although several devices are commercially available to verify the dose distributions delivered in external radiotherapy treatments, the options available to perform quality control of the HDR brachytherapy treatment process are more limited [2]. The usual procedure in this field is to use well-type chambers to determine the RAKR. Nevertheless, there is not a clear consensus regarding techniques to verify the dose distribution in these treatments [3].

Dose measurements in brachytherapy are especially challenging due to the steep dose gradients and small distances involved, where order of magnitude variations can be found in clinical regions [4]. On the other hand, although the tendency is to use algorithms that consider patient heterogeneities, in general, the process through which the delivered dose is calculated is relatively simple in comparison with the external radiotherapy field since all the material medium is considered as water [5].

There are several ways to perform a dosimetric evaluation in a brachytherapy audit. The most elementary one is to measure the RAKR with a well-type chamber, as done by Carlsson et al. [6]. However, this method does not provide any information about the spatial dose distribution. Another possibility is to place an ionization chamber at a fixed distance from the source and perform a dose measurement. Herreros et al. [7] presented a phantom that consists of a polymethyl metacrylate (PMMA) cylinder with 4 equally spaced tunnels to insert the needles and, in the middle, an insert to hold either a scintillation detector or a Farmer chamber. Higher spatial resolution can be achieved by placing a dosimeter, either thermoluminescent (TLD) [8] or optically stimulated luminescent dosimeter (OSLD) [9], instead of a chamber. However, in this case an instantaneous measurement of the dose is not obtained, being necessary to read the dosimeter a posteriori. The Quality Assurance Network for Radiotherapy (EQUAL) of the European Society for Radiotherapy and Oncology (ESTRO) offers a service for HDR and pulsed dose rate (PDR) brachytherapy audits for systems using Iridium-192 (^192^Ir) sources, available to users since 2004. The dose is measured using a phantom with TLDs merged in water [8], whereas the image reconstruction can be evaluated using the Baltas phantom [10,11]. An alternative dosimeter with a higher spatial resolution is radiochromic films. Palmer et al. [12,13] introduced both radiochromic films and the gynecological applicator in a phantom designed for this purpose. This entire set is submerged in water.

In brachytherapy, the uncertainty in the delivered dose is higher than in the external radiotherapy field. This fact results in a higher acceptance level for the deviation between the expected and the measured dose. For instance, in the EQUAL-ESTRO brachytherapy audit, a deviation below 7% is considered an optimal result, whereas this value would be 3% for the external radiotherapy audit [3].

The audits cited above are for HDR or PDR brachytherapy. To a lesser extent, there are also dosimetric audits for electronic brachytherapy, as is the case of Palmer et al. [14]. However, to our best knowledge, the audits in the field of low dose rate (LDR) implants with ^125^I seeds are limited to center intercomparisons, as reported by Palmer et al. [15], where calibrated seeds were mailed to six different centers and the determined RAKR by each one using its own well-type chamber was compared.

Another aspect of auditing in the brachytherapy treatments is the image reconstruction. Due to the steep dose gradient, the image reconstruction of the dwell positions and prescription points has an important influence in the final precision of the clinical dosimetry [16]. The EQUAL laboratory offers a phantom to evaluate the image reconstruction acquired with CT [11]. Nevertheless, in brachytherapy, other image modalities are also used. Ultrasound (US) images are used in prostate implants; whereas, in gynecological treatments, magnetic resonance images (MRI) provide better tissue contrast. Doyle et al. [17] gather recommended phantom and tests by different entities for US systems used in prostate treatments, whereas recommendations for the commissioning, quality control and considerations for MRI acquisitions in HDR brachytherapy can be found in the American Association of Physicists in Medicine (AAPM) TG-303 [18]. On the other hand, recent publications also provide methodologies to verify the Treatment Planning System (TPS) or for treatment verification in HDR brachytherapy [19,20].

Despite the examples cited above, the number of entities that offer the service of dosimetric audits in brachytherapy is reduced in comparison to the external radiotherapy field [4]. Although external dosimetric audit is recommended in many authoritative documents [3], currently, it is not a common practice in brachytherapy. Recently, the International Atomic Energy Agency (IAEA) launched a Coordinated Research Project (CRP E24023) with the aim of developing a methodology to perform HDR brachytherapy audits, as well as encourage the development of national dosimetric audit networks in the Member States.

In this context, the objective of this work is to implement a methodology to perform dosimetric audits in HDR brachytherapy. The requirements that this system must meet are the following: able to be mailed (so it can be used to perform dosimetric audits on-site), user-friendly and valid for systems using either ^192^Ir or Cobalt-60 (^60^Co) sources. The criteria and studies performed for its design, together with the in-house measurements for its characterization, are presented in the following.

## 2. Materials and Methods

### 2.1. Dosimetry System

**Figure 1:**
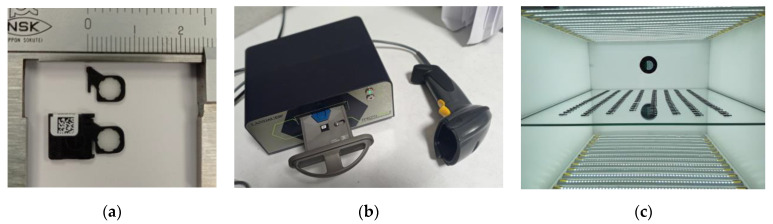
(**a**) The nanoDot OSLD from Landauer [21]. (**b**) The microSTARii reader from Landauer [21]. (**c**) A homemade eraser consisting of multiple rows of LEDs for a more exhaustive annealing of the OSLDs.

The detectors used were optically stimulated luminescent (OSL) nanoDot dosimeters (Nagase Landauer, Ltd., Tsukuba, Japan). These dosimeters consist of a 5 mm diameter and 0.15 mm thickness cylinder of Al_2_O_3_:C inserted in a 10 × 10 × 2 mm^3^ plastic box (Figure 1a). The readings are performed with a microSTARii reader (Nagase Landauer, Ltd., Tsukuba, Japan), which works with a high-intensity pulsed light-emitting diode (LED) that induces the luminescence in the material (Figure 1b). A home-made eraser is used for a more exhaustive annealing. It consists of multiple rows of LEDs of 6500 K temperature color where the dosimeters are exposed for 4 h (Figure 1c).

This system is placed at the Spanish Centro Nacional de Dosimetría (CND) and was previously studied by Pujades et al. [21] for dosimetric audits of external radiotherapy beams, where the dose linearity, reproducibility, loss of signal at each reading, energy dependence and fading were characterized. These results were reused for this work, except the part corresponding to the energy dependence factor since, as explained in the following sections, it is included in the calibration coefficient.

For this work, the individual sensitivity factor (ISF) was calculated for a batch of 100 OSLDs. For its characterization, all the dosimeters were irradiated to a dose of 1 Gy at the Radiations Physics Laboratory (RPL) of the Universidad Santiago de Compostela (USC), an accredited Secondary Standard Dosimetry Laboratory. Then, the ISF was calculated with respect to the average sensitivity of the batch.

### 2.2. Phantom Design

The first goal in the phantom design was to look for a configuration that led to a homogeneous dose distribution in the area where the nanoDot is placed and, simultaneously, with dimensions small enough to represent the typical distances of the treatments in this clinical practice. With this purpose, tests were performed with the TPS Oncentra^®^ Brachy from Elekta AB (Stockholm, Sweden) for ^192^Ir, and with the TPS SagiPlan^®^ from BEBIG Medical GmbH (Berlin, Germany) for ^60^Co.

**Figure 2:**
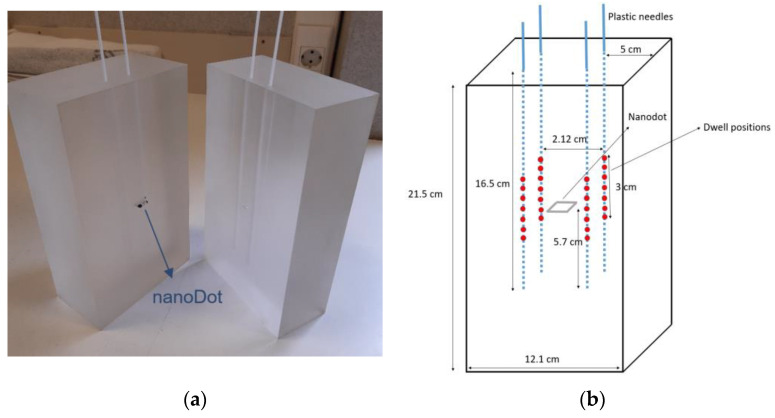
(**a**) Phantom: It consists of a PMMA parallelepiped with four slots to insert the plastic needles and a slot to hold the OSL nanoDot dosimeter. (**b**) Dimensions of the phantom. The red spots are the dwell positions, equally spaced 0.5 cm.

The phantom consists of a PMMA parallelepiped with four slots to insert the plastic needles that are connected to the brachytherapy afterloader (Figure 2a). Its dimensions are shown in Figure 2b. Inside the phantom, there is a slot to hold the OSL nanoDot dosimeters.

### 2.3. Calibration of the System

**Figure 3:**
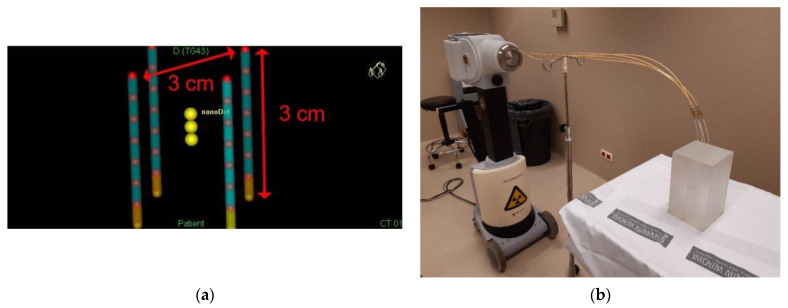
(**a**) Reference plan with 7 active positions (red spheres) per catheter equally spaced 5 mm. (**b**) Irradiation set-up. The four plastic needles inserted in the phantom are connected to the afterloader. The dosimeter is placed inside the phantom.

In this set-up, the dosimeter is irradiated with multiple angulations and from different distances to the source. Therefore, if the dosimeter was calibrated in a laboratory using, for instance, a ^60^Co beam with normal incidence, multiple angular dependence factors should be ideally applied and characterized. In addition, there may be a distance dependence in the photon spectrum reaching the nanoDot. Therefore, different energy factors should also be characterized and applied. To simplify this, we opted to calibrate with the same irradiation set-up, as the one used to perform the audit in the hospitals, which is shown in Figure 3. With this methodology, the energy-angular conditions are the same in the calibration and in the audit exercise, so the energy-angular dependence is already included in the calibration coefficient.

Two hospitals were established as reference hospitals: the Hospital Universitari i Politècnic La Fe, with a MicroSelectron V2 from Elekta for ^192^Ir, and the Hospital Universitari Sant Joan d’Alacant, with a MultiSource from BEBIG for ^60^Co. Both reference hospitals follow the QA/QC protocol described in ESTRO Booklet No.8 [3]. Their TPS strictly follow the ESTRO-AAPM recommendations for High Energy Brachytherapy Source Dosimetry (HEBD) [22], including the consensus recommended data, which have been thoroughly verified prior to this project. Besides this, these centers were chosen because of their proximity to the CND, where the dosimetry system is placed while also being regional reference hospitals.

A reference plan is designed, consisting of 7 active positions per catheter, equally spaced 5 mm, so that the diagonal and the height of the cube are both 3 cm (Figure 3a) and with the same irradiation time for all the dwell positions. With this configuration, a dose of 3 Gy to the point where the dosimeter is located is prescribed in the TPS, i.e., in the TG-43 conditions [5]. Dosimeters are irradiated with this methodology in the reference hospitals (Figure 3b) and then read with the microSTARii reader (Figure 1b). The relation between the dosimeter signal and the prescribed dose in the TPS is established as the calibration coefficient. If ${\overset{-}{L}}_{c}$ is the average reading of the calibration dosimeters and D_TG43_ is the dose prescribed in the TPS, the calibration coefficient remains as ${F=D_{TG43}/\overset{-}{L}}_{c}$.

### 2.4. Characterization of the Scatter Conditions

**Figure 4:**
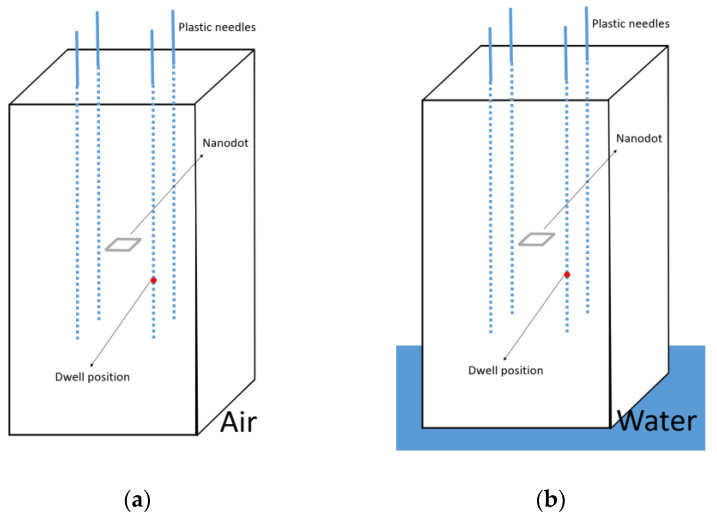
Configuration for the MC simulations to characterize the scatter conditions in the irradiation set-up. Air was selected as the surface material (**a**), and then it was replaced by water (**b**).

There are 5 cm of PMMA in the lateral direction from the source to the phantom surface (Figure 2b). According to Granero et al. [23], with these dimensions, the conditions should be close to full scatter conditions. Even so, according to the recommendations given in the ESTRO Booklet No.8 [3], to maintain the contribution of scattered radiation at a minimum, the phantom should be placed at least 1 m from any wall. With this procedure, if the phantom is always placed in the position shown in Figure 3, surrounded by air, the only factor that can vary between the irradiation between hospitals and the calibration conditions is the surface material on which the phantom is supported during the irradiations. To study how these differences may affect the absorbed dose in the nanoDot, Monte Carlo (MC) simulations were performed with the PENELOPE/Peneasy software for both ^192^Ir and ^60^Co sources. The simulations were performed in the most unfavorable case, namely, for the source position closest to the surface (Figure 4). Air was selected as the material for the surface (Figure 4a), and then, it was replaced by water (Figure 4b). The absorbed dose in the nanoDot for both cases was compared.

The simulations were performed following the recommendations of Perez-Calatayud et al. [22] for the spectra, compositions and dimensions of the brachytherapy sources. The plastic needles are modeled as hollow cylinders with walls of 0.3 mm thickness made of tissue-equivalent plastic. The phantom was modeled as a PMMA parallelepiped, whose dimensions are shown in Figure 2b. The external box containing the sensitive material of the nanoDot was modeled as a plastic box with dimensions of 10 × 10 × 2 mm^3^. The sensitive area was modeled as a 5 mm diameter and 0.2 mm height cylinder. This area is placed, as illustrated in Figure 2b, in the center of the cube formed by the active dwell positions. The sensitive material, Al_2_O_3_, was generated with the Penelope material library with a density of 1.41g/cm^3^ [24].

To establish whether there is a statistically significant difference between the two cases illustrated in Figure 4, the *z*-test is applied for the null hypothesis of equality of two distributions [25]. The z value is computed as:

(1) $$z=\frac{\mu_{1}-\mu_{2}}{\sqrt{\sigma_{1}^{2}+\sigma_{2}^{2}}}$$

where μ and σ are the mean and the standard deviation respectively of the two distributions that are being compared. Both distributions are statistically different for a signification level α = 0.01 if it is fulfilled that *z* ≥ 2.58.

### 2.5. Study of the Spectra Reaching the nanoDot for Different ^192^Ir Source Models

MC simulations were also performed to study differences in the photon spectra for different types of ^192^Ir sources and how these can affect the nanoDot response. Microselectron V2, Flexisource, Varisource VS2000 and Ir2.A85-2 source models were simulated with the Penelope/Peneasy software. The spectra, compositions and dimensions were taken from the recommendations of Perez-Calatayud et al. [22]. In the simulations, the source models were placed in the different source positions of our irradiation set-up (see Figure 2b), and the photon spectra and the mean photon energy reaching the nanoDot in each case were compared. Since there are only two types of ^60^Co sources commercially available and, to our knowledge, the most used is the one considered in this work, this comparison was only performed for the different ^192^Ir source models.

### 2.6. Dwell Times

**Figure 5:**
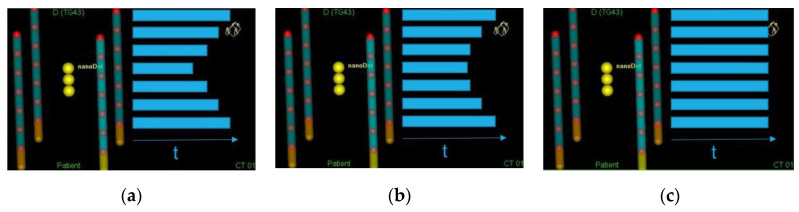
(**a**–**c**) are examples of dwell time distributions that would lead to a uniform dose distribution, with a homogeneity below 2%, in the area where the nanoDot is placed in the irradiation set-up. To avoid differences in the dose measurement due to the angular dependence of the anodot, the same dwell time was fixed for all the dwell positions (**c**).

For the irradiation set-up described, with 4 catheters and 7 active positions per catheter, there are multiple configurations in the dwell time distribution which would lead to a uniform dose distribution in the volume where the nanoDot is placed. One possibility was to give instructions to the audited hospitals for optimizing the dwell times to get a uniform dose distribution in the nanoDot location. However, this could lead to the following situation: a hospital obtaining a time distribution as the one shown in Figure 5a and another hospital obtaining a time distribution slightly different, such as the one shown in Figure 5b. This would lead to the dosimeter being irradiated in different proportions under different angles. According to Cruz et al. [26], these nanoDot dosimeters show an angular dependence of up to 29% for an energy of 40 keV and up to 6% for an energy of 1500 keV. Therefore, differences in the dwell time distribution would increase the uncertainty in the dose measurement. To avoid this situation, the same time was set for all the dwell positions (Figure 5c).

### 2.7. Stability of the Reader

According to the manufacturer and our own experience, the stability of the microSTARii reader can vary around 5% in a period of 24 h. To minimize the uncertainty introduced due to this effect, control dosimeters were irradiated at the RPL of the USC to a dose of 2 Gy and read together with the calibration dosimeters. The average reading of these control dosimeters (${\overset{-}{C}}_{c}$_)_ is taken as a reference.

Every time a dosimetry audit is performed, new control dosimeters are irradiated to a dose of 2 Gy and read together with the dosimeters irradiated in the audited centers. Then, the readings of the audit centers are corrected with a stability factor $C={\overset{-}{C}}_{c}/{\overset{-}{C}}_{i}$, where ${\overset{-}{C}}_{i}$ is the average reading of the new control dosimeters.

### 2.8. Final Dose Evaluation

The dose value obtained in an audit exercise can then be expressed as:

(2) $$D=FC{\overset{-}{L}}_{i}$$

where *F* is the calibration coefficient, *C* the stability factor and ${\overset{-}{L}}_{i}$ the average reading of the dosimeters irradiated in the audited center.

## 3. Results

### 3.1. Dose Distribution

**Figure 6:**
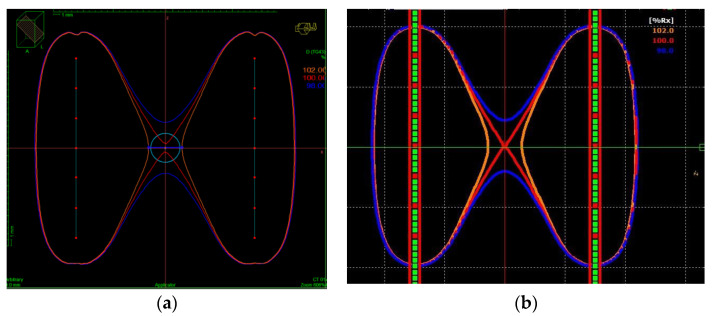
Dose distribution in the irradiation set-up for ^192^Ir (**a**) and for ^60^Co (**b**). The vertical lines are two catheters located at opposite corners of the cube (see Figure 3a). The blue, red and orange lines correspond to the 98%, 100% and 102% isodose lines, respectively.

The dose distribution in the TPS is shown for ^192^Ir and ^60^Co in Figure 6a,b, respectively. It is shown in a plane that contains two catheters located at opposite corners of the cube, which is separated 3 cm (see Figure 3a). The dose distribution is similar for both sources. In both cases, the isodoses of 98% and 102% are separated, at least 0.82 cm and 0.57 cm, respectively. This means that the sensitive area of the nanoDot, which is placed in the center of this cube, is contained in an area where the homogeneity of the dose distribution is 2%.

### 3.2. Calibration Coefficients

The calibration coefficients obtained with the described methodology and for a prescribed dose of 3 Gy in the center of the nanoDot are:

$$F_{Ir}=\left(3.63\pm 0.07\right)10^{-5}{Gy}_{TG43}/counts$$

$$F_{Co}=\left(3.58\pm 0.07\right)10^{-5}{Gy}_{TG43}/counts$$

for ^192^Ir and ^60^Co respectively.

### 3.3. Characterization of the Scatter Conditions

For the ^192^Ir source, 4.6 × 10^10^ events were simulated for cases (a) and (b) of Figure 4. The statistical uncertainty in the simulation was 0.07% (*k = 2*). For the ^60^Co source 10 × 10^11^ events were simulated. In this case, the uncertainty in the simulation was 0.09% (*k = 2*). The differences in the absorbed dose in the nanoDot due to the support material were 0.10% for ^192^Ir and 0.014% for ^60^Co.

The *z*-test was calculated as described in Equation (1). The *z* value obtained in the comparison of the ^192^Ir simulations was *z_Ir_* = 2.08, whereas for the ^60^Co simulations was *z_Co_* = 0.23. According to Daniel et al. [25], both distributions are statistically different for a signification level α = 0.01 if it is fulfilled that z ≥ 2.58. Therefore, no statistically significant differences are found in the absorbed dose in the nanoDot between situations (a) and (b) illustrated in Figure 4, neither for ^192^Ir nor for ^60^Co.

### 3.4. Study of the Spectra Reaching the nanoDot for Different ^192^Ir Source Models

**Figure 7:**
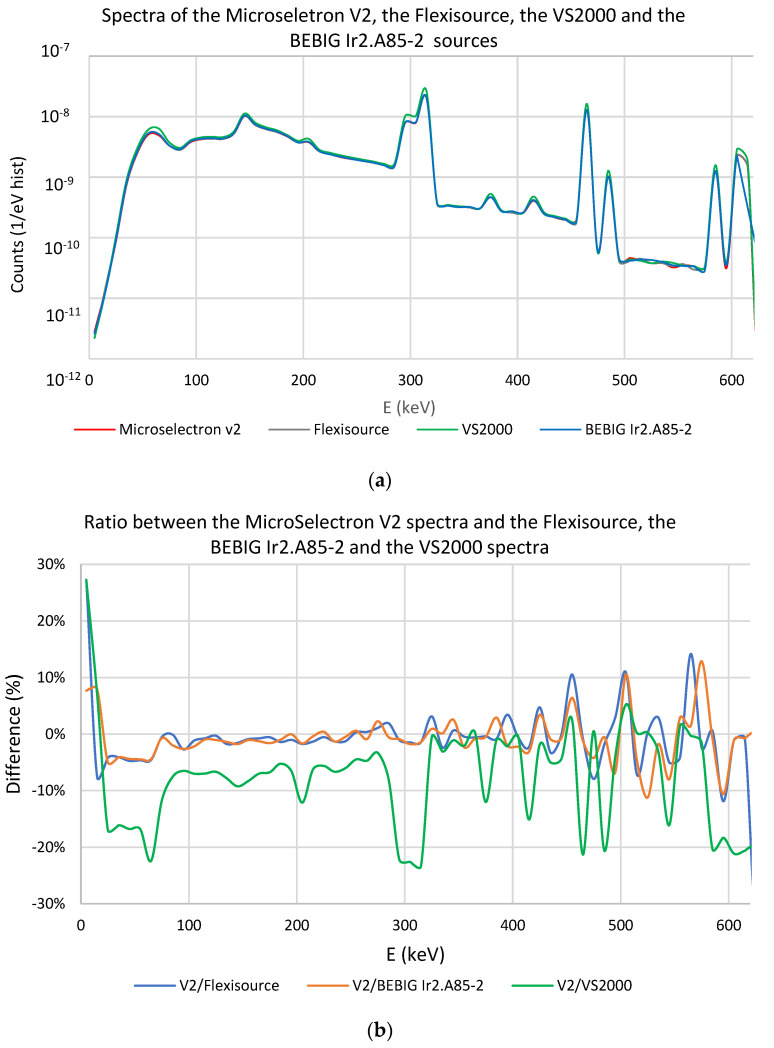
(**a**) Photon spectra reaching the sensitive volume for the MicroSelectron V2, the Flexisource, the VS2000 and the BEBIG Ir2.A85-2 sources. (**b**) Relative difference between the photon spectra reaching the sensitive volume for the MicroSelectron V2 and the rest of the source models. The source to nanoDot distance is 1.5 cm.

Figure 7a shows the spectra of the MicroSelectron V2, the Flexisource, the VS2000, and the Ir2.A85-2 sources, whereas Figure 7b shows the relative difference between the MicroSelectron V2 photon spectrum and the rest of these source models. The distance between the nanoDot and the dwell position is 1.5 cm. The results show differences below 5% between the MicroSelectron V2 and the Flexisource models in most parts of the spectra. It can also be observed that, for the energy of maximum emission (around 315 keV), differences between the V2 and the Flexisource models, or between the V2 and the Ir2.A85-2 models, are below 2%. However, this is not the case for the VS2000 source, where differences over 20% with respect to the V2 can be observed.

Additionally, a dependency on the distance between the dwell position and the nanoDot is observed in the spectra reaching the detector. Table 1 shows the mean energy of the spectra reaching the nanoDot for the different source-nanoDot distances in the irradiation set-up for the Microselectron V2, the Flexisource, the Varisource VS2000 and the BEBIG Ir2.A85-2 models. The deviation between the mean energy of each source model and the mean energy of the Microselectron V2 is shown in Table 2. It is observed that, for a coverage factor of *k* = 1, all the deviations are compatible with zero except the one corresponding to the VS2000. For this case, higher differences in the mean energy in comparison with the MicroSelectron V2 are observed for distances nearer to the source.

### 3.5. Estimated Uncertainty in the Dose Measurement

The total uncertainty in the dose measurement is 3.0% (*k* = 1) for ^60^Co sources and for the MicroSelectron V2, Flexisource and BEBIG ^192^Ir sources. Due to the differences found in the photon spectra reaching the detector for the Varisource VS2000 model, the uncertainty was increased in this case to 3.1% (*k* = 1) to allow for differences due to the OSLD nanoDot response. The following uncertainties, summarized in Table 3, were considered:

- Uncertainty in the dosimeter reading (*L*): The estimated uncertainty in the dosimeter reading is 0.5 % (*k* = 1), considering the uncertainty due to fading, depletion factor, and characterization of the ISF.
- Uncertainty in the reader stability (*C*): An uncertainty of 1.3% (*k* = 1) was estimated for the stability of the reader. This uncertainty was reduced from 5% (see Section 2.7) to 1.3% using the control dosimeters described above.
- Uncertainty in the RAKR: This was taken from the calibration certificate of the source, being 1.7% (*k* = 1) for both ^60^Co and ^192^Ir sources.
- Uncertainty in the dwell position: The phantom is designed to insert 6F needles, which have 2 mm of external diameter. Considering the dwell thickness, a dwell shift in the direction of the catheter below 1 mm (according to the tolerance established in the quality protocols) and the dose homogeneity in the area where the nanoDot is placed leads to an estimated uncertainty of 0.7% (*k* = 1).
- Uncertainty in the irradiation time: Considering that there are 7 active positions per catheter and a time resolution of 0.1 s/position, the estimated uncertainty is 0.1% (*k* = 1).
- Uncertainty in the OSLD response due to differences in the spectra of the VS2000 ^192^Ir source: Our results show a mean photon energy reaching the nano-Dot for the Varisource VS2000 model of 240.6 keV to be compared with the 234.6 keV found for the MicroSelectron V2 source. If these data are contrasted with the energy dependence reported by Cruz et al. [26], considering the angular incidences of our irradiation set-up, the variation in the nanoDot response due to this energy variation would be between 0.1% and 0.3%. Therefore, a conservative uncertainty of 0.3% (*k* = 1) is considered.

**Table 3:** Relative standard uncertainty in the dose measurement.

Components	Uncertainty (%) (*k* = 1)		
	^192^Ir (V2, Flexisource and BEBIG)	^192^Ir (VS2000)	^60^Co
OSLD reading (*L*)	0.5	0.5	0.5
Reader stability (*C*)	1.3	1.3	1.3
RAKR	1.7	1.7	1.7
Dwell position	0.7	0.7	0. 7
Irradiation time	0.1	0.1	0.1
Calibration coefficient (*F*)	1.9	1.9	2.0
OSLD response		0.3	
Overall uncertainty	3.0	3.1	3.0

## 4. Discussion

This study presents a user-friendly phantom to perform external dosimetric audits of HDR brachytherapy systems. Results of the MC simulations for the characterization of the scatter conditions indicate that the surface material on which the phantom is supported during the irradiation does not affect the absorbed dose in the nanoDot. This is the only factor that can vary between the irradiation from one hospital to another one or with respect to the calibration set-up. Although in the lateral direction, where the phantom is surrounded by air, there would not be full scatter conditions, these conditions are the same both in the calibration and in the audit measurement, so that they would be directly incorporated into the calibration coefficient. Therefore, it is not necessary to give instructions to the audited center about the surface material on which the phantom is supported during the irradiations. The only requirement is that the phantom must be placed vertically.

As the system is calibrated according to the TG-43, the audit is valid for hospitals using this formalism. If the formalism is updated and the values used are modified, the calibration factors should also be updated. Therefore, it must be confirmed that the audited hospitals use the consensus values provided in the Joint AAPM/Imaging and Radiation Oncology Core (IROC) and Brachytherapy Physics Quality Assurance System (BRAPHYQS) repositories.

In this work, the dose distribution is evaluated in a TPS following the TG-43 formalism [5]. In this irradiation set-up, the medium considered is water instead of PMMA. The use of PMMA as an equivalence to water to evaluate the dose distribution has been previously studied from kilovoltage to megavoltage photon beams [27,28,29]. Results show that dose distribution evaluated in PMMA is in good agreement with those evaluated in water. Meli et al. [30] reported the ratios of mass-energy absorption coefficients between water and PMMA for different photon energies and phantom sizes. Their results show that these coefficients are dependent on the phantom dimensions and on the distance to the source. According to their results, for a 10 cm radius phantom, and considering the dimensions of the nanoDot (5 mm diameter and 0.15 mm height), the variation in the ratio between the absorbed dose between water and PMMA should be less than 0.2% along the area of the nanoDot in our irradiation set-up. Therefore, disregarding differences in the absolute absorbed dose (below 4% according to the reported results) and attending only to the relative dose distribution, the assumption that the sensitive area of the dosimeter is contained in an area where the homogeneity of the dose distribution is 2% can be extended from the TPS to our irradiation set-up.

Cruz et al. [26] reported the energy dependence of the nanoDot dosimeters in nuclear medicine regions using MC simulations. According to their results, the dosimeter response should be around 5% higher for the case of ^192^Ir in comparison with ^60^Co. However, attending to our calibration coefficient results, our dosimeter reading is 1% lower for ^192^Ir than for ^60^Co. This could be due to the fact that, in our configuration set-up, the dosimeter is being irradiated under different angles (from 90° to 45°). Therefore, the angular dependence also comes into play. According to the data reported by Cruz et al. [26], a higher angular dependency for lower energies is faced, so the dosimeter response for the different incidences in our irradiation set-up should be lower for ^192^Ir than for ^60^Co. This fact could compensate the energy dependency effect, obtaining, in our case, a response slightly higher for ^60^Co than for ^192^Ir.

Regarding the differences in the mean photon energy spectra reaching the nanoDot, for the MicroSelectron V2, the Flexisource and the BEBIG ^192^Ir sources, no significant differences are observed in our results that can lead to differences in the nanoDot response due to the energy dependence. However, this is not the case for the Varisourse VS2000, where, attending to the data reported by Cruz et al. [26], the maximum difference in the nanoDot response due to these differences is estimated as 0.3%. If this difference is considered as an uncertainty in the dose measurement, the calibration coefficient can also be extended for this source model.

## 5. Conclusions

In this work, it is presented a user-friendly system valid to perform mailed dosimetric audits in HDR brachytherapy for systems using either ^192^Ir or ^60^Co sources. The calibration coefficients and the uncertainty in the dose measurement were characterized. The results in the study of the spectra for the different ^192^Ir source models indicate that no significant differences are observed between the spectra of the MicroSelectron V2, the Flexisource and the BEBIG ^192^Ir sources. However, for the Varisource VS2000, a higher uncertainty is considered in the dose measurement to allow for the OSLD nanoDot response.

## Figures and Tables

**Table 1:** Mean energy (keV) of the photon spectra reaching the nanoDot for the different source to nanoDot distances in the irradiation set-up for the different ^192^Ir sources. The uncertainty is expressed with a coverage factor of *k* = 1.

	Source to NanoDot Distance (cm)			
Source Model	2.12	1.80	1.58	1.50
V2	300.1 ± 0.9	300.0 ± 0.9	287.2 ± 0.9	234.6 ± 0.7
Flexisource	299.4 ± 0.9	299.2 ± 0.9	286.4 ± 0.9	233.9 ± 0.7
VS2000	296.9 ± 0.9	296.4 ± 0.9	283.1 ± 0.9	240.6 ± 0.7
BEBIG	299.3 ± 0.9	299.4 ± 0.9	286.3 ± 0.9	233.9 ± 0.7

**Table 2:** Deviation between the mean energy of each ^192^Ir source model and the MicroSelectron V2 model for the different source to nanoDot distances in the irradiation set-up. The uncertainty is expressed with a coverage factor of *k* = 1.

	Source to NanoDot Distance (cm)			
Source Model	2.12	1.80	1.58	1.50
Flexisource/V2	−0.3 ± 0.4%	−0.3 ± 0.4%	−0.3 ± 0.4%	−0.3 ± 0.4%
VS2000/V2	−1.1 ± 0.4%	−1.2 ± 0.4%	−1.4 ± 0.4%	2.6 ± 0.4%
BEBIG/V2	−0.3 ± 0.4%	−0.2 ± 0.4%	−0.3 ± 0.4%	−0.3 ± 0.4%

## Data Availability

The data that support the findings of this study are available from the corresponding author, Oliver-Cañamás L., upon reasonable request.
